# A New Asynchronous RTK Method to Mitigate Base Station Observation Outages

**DOI:** 10.3390/s19153376

**Published:** 2019-08-01

**Authors:** Yuan Du, Guanwen Huang, Qin Zhang, Yang Gao, Yuting Gao

**Affiliations:** 1 College of Geology Engineering and Geomantic, Chang’an University, 126 Yanta Road, Xi’an 710054, China; 2 Department of Geomatics Engineering, University of Calgary, Calgary, AB T2N 1N4, Canada

**Keywords:** GNSS RTK, base station, observation outages, ARTK, asynchronous errors

## Abstract

Real-time kinematic (RTK) positioning is a satellite navigation technique that is widely used to enhance the precision of position data obtained from global navigation satellite systems (GNSS). This technique can reduce or eliminate significant correlation errors via the enhancement of the base station observation data. However, observations received by the base station are often interrupted, delayed, and/or discontinuous, and in the absence of base station observation data the corresponding positioning accuracy of a rover declines rapidly. With the strategies proposed till date, the positioning accuracy can only be maintained at the centimeter-level for a short span of time, no more than three min. To address this, a novel asynchronous RTK method (that addresses asynchronous errors) that can bridge significant gaps in the observations at the base station is proposed. First, satellite clock and orbital errors are eliminated using the products of the final precise ephemeris during post-processing or the ultra-rapid precise ephemeris during real-time processing. Then the tropospheric error is corrected using the Saastamoinen model and the asynchronous ionospheric delay is corrected using the carrier phase measurements from the rover receiver. Finally, a straightforward first-degree polynomial function is used to predict the residual asynchronous error. Experimental results demonstrate that the proposed approach can achieve centimeter-level accuracy for as long as 15 min during interruptions in both real-time and post-processing scenarios, and that the accuracy of the real-time scheme can be maintained for 15 min even when a large systematic error is projected in the U direction.

## 1. Introduction

The real-time kinematic (RTK) positioning technique enables high-precision navigation and positioning using global navigation satellite systems (GNSS) as it can quickly fix double-differenced carrier phase ambiguities in open and unobstructed environments. This technique is currently used in both real-time and post-mission scenarios to support a wide range of applications, including landslide hazard, building monitoring, and measuring tides and ocean waves in coastal areas [1,2,3,4,5]. In GNSS real-time deformation monitoring, the base station and monitoring station are generally in an automatic unattended state. A reference station often provides data services for a dozen or more monitoring stations to better study the characteristics of the deformation body even in a small area. Once the reference station data is interrupted, the recovery time is often dozens of minutes or even an hour. Therefore, ensuring the continuity and reliability of the base station data is very important. Continuous monitoring is essential to studying the kinematics and to predicting the behavior of monitored object [6,7,8]. Furthermore, continuous and reliable RTK positioning relies on the real-time availability of GNSS observations at the base station to effectively reduce or eliminate spatially correlated errors and subsequently fix the ambiguity parameters [9]. The classic synchronous RTK (SRTK) technique requires a pair of valid observations from the same satellite to be obtained simultaneously from both the base station and the rover station, as the positioning accuracy of a rover receiver can be at the centimeter or sub-millimeter level. One of the essential requirements of RTK positioning is the continuous availability of base station observations. Thus, outages of base station observations caused by the failure of communications links under challenging environments are a concern, particularly for events such as landslides and building construction, which need continuous monitoring [1,2,3,4,5,6,7,8,9,10]. Outages of base station observations break that continuity; therefore, it is of great importance that the positioning accuracy of the rover is maintained even during gaps in the base stations observations.

Various methods have been proposed to mitigate gaps in the base station observations so that continuous RTK solutions can be provided during outage periods [11,12,13,14,15,16]. For example, an algorithm was proposed to estimate the rover position based on time-differenced carrier observations [12]. Although it was stated that the proposed algorithm was a single-difference model, this method employed the difference between the satellites to eliminate the receiver clock difference and the position of the rover was determined based on the time difference between the epochs received by the rover. The algorithm achieved a positioning accuracy of approximately 5 cm within 1 min of the gap in the data provided by the base receiver [12]. However, this is a post-processing method and the poorest precision was observed at the midpoint of the duration for which the data were missing; this is consistent with the error propagation theorem for iterative calculations. Lawrence (1999) proposed a reference carrier phase prediction method for kinematic situations in which the reference phase prediction error was in the decimeter range after 10 s based on observational data from 7 s in the past [14]. Zhang et al. (2015) proposed an asynchronous RTK (ARTK) positioning method that utilized direct asynchronous double-difference (DD) observations between receivers and satellites and provided centimeter-level accuracy when there was a 15 s gap in the base station observations. However, instead of eliminating errors such as ionospheric delay and residual asynchronous error, this the method simply ignored those [13].

A common limitation of the aforementioned methods is that the positioning accuracy decreases as the gap in the observations from the base station increases. Moreover, the centimeter-level positioning accuracy is available only for a short period of time (no more than three min) when the observations of the base station are not available for the current epoch. Thus, this study proposes a new asynchronous RTK (ARTK) method to reduce the impact of asynchronous errors arising because of the gaps in base station observations. Further, although Zhang et al. (2015) found that an asynchronous error within 15 s could be tolerated by the ARTK method, the asynchronous error that occurs with an increase in time needs to be investigated [13]. Therefore, the first step in this study was to implement an asynchronous error strategy to maintain the positioning service during outages of the base station observations. Then, the satellite clock and orbital errors are eliminated using the final precise ephemeris in the case of post-mission data processing or the ultra-rapid precise ephemeris for real-time applications [17]. In addition, the tropospheric error is corrected using the Saastamoinen model, while the asynchronous ionospheric delay is corrected using carrier phase measurements of the rover receiver. Finally, a straightforward first-degree polynomial function is employed to predict the residual asynchronous error. The processing of the asynchronous error based on the proposed method is able to maintain the positioning accuracy for a longer period of time than that possible with existing algorithms when there are gaps in the base receiver observations.

The objectives of this work include the study of GNSS asynchronous error theory and the development of a correction strategy to maintain the RTK positioning at centimeter-level accuracy during the outages of the base station observations. The remainder of this paper is organized as follows. First, the methodology of the proposed ARTK model is derived. Then, the results of experiments in static and dynamic positioning that validate the proposed method are presented. In the experiments, post-processing was implemented using final precise ephemeris products and real-time processing utilized ultra-rapid precise ephemeris products. Finally, the conclusions drawn from this study are presented in the last section.

## 2. Methodology 

In this section, the implementation details of the proposed ARTK are provided. First, the ARTK model is derived from classical RTK models and the associated asynchronous errors are derived from the asynchronous data difference. Then the most influential terms in the asynchronous ionospheric delay and residual error are further analyzed. Finally, the new ARTK strategy is presented.

### 2.1. Real-Time Kinematics (RTK) and Asynchronous Real-Time Kinematics (ARTK)

The undifferenced carrier-phase observation equation relating satellites *i* and base receiver *B* is as follows [13,18]:

(1) $$\begin{array}{ll} \varphi_{B}^{i}\left(t_{0}\right)=R_{B}^{i}\left(t_{0}\right) & +\lambda N_{B}^{i}\left(t_{0}\right)+c\left[\delta t_{B}\left(t_{0}\right)-\delta t^{i}\left(T_{0}\right)\right]-I_{B}^{i}\left(t_{0}\right)+T_{B}^{i}\left(t_{0}\right)+\delta \varphi_{B}\left(t_{0}\right)\\ & -\delta \varphi^{i}\left(t_{0}\right)+E^{i}\left(t_{0}\right)+\varepsilon_{B}^{i}, \end{array}$$

where φ is the carrier phase measurement, $t_{0}$ is the signal arrival time, $T_{0}$ is the corresponding emission time, R is the geometric distance, λ is the signal wavelength, N is the carrier phase ambiguity in cycles, c is the velocity of light, $\delta t_{B}$ is the receiver clock error, $\delta t^{i}$ is the satellite clock error, I is the ionospheric delay, T is the tropospheric delay, $\delta \varphi_{B}$ is the initial phase bias in the carrier phase measurement introduced by the receive antenna, $\delta \varphi^{i}$ is the initial phase bias in the carrier phase measurement introduced by the satellite antenna, E is the ephemeris error, and ε is the receiver noise in the carrier phase [19,20].

(2) $$\varphi_{BA}^{ij}\left(t_{1},t_{1}\right)=R_{BA}^{ij}\left(T_{1},T_{1}\right)+\lambda N_{BA}^{ij}\left(t_{1},t_{1}\right)+\varepsilon_{BA}^{ij}\left(t_{1},t_{1}\right)$$

However, in the following asynchronous DD observation model, some correlation errors cannot be eliminated:

(3) $$\begin{array}{ll} \varphi_{BA}^{ij}\left(t_{0},t_{1}\right)= & R_{BA}^{ij}\left(T_{0},T_{1}\right)+\lambda N_{BA}^{ij}\left(t_{0},t_{1}\right)+c\delta t^{ij}\left(T_{0},T_{1}\right)+E^{ij}\left(T_{0},T_{1}\right)+T_{BA}^{ij}\left(t_{0},t_{1}\right)\\ & \;-I_{BA}^{ij}\left(t_{0},t_{1}\right)+\varepsilon_{BA}^{ij}\left(t_{0},t_{1}\right). \end{array}$$

**Figure 1:**
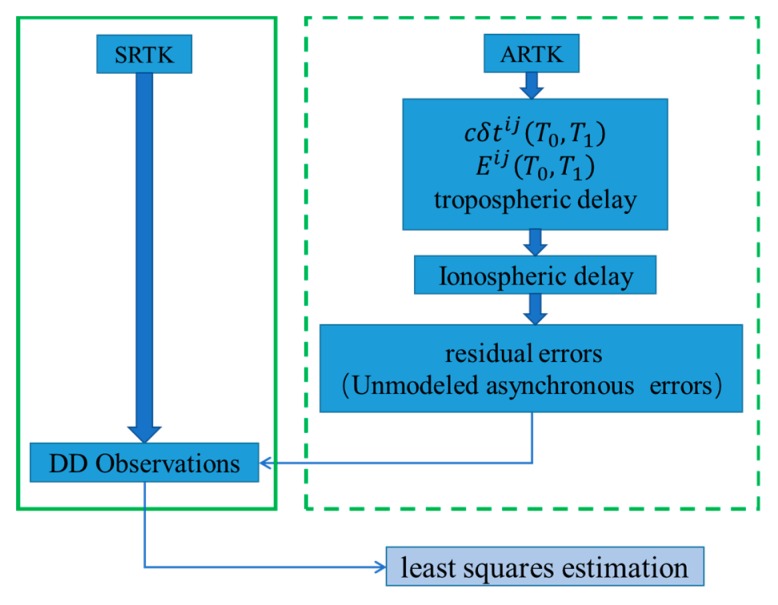
Different processes used in synchronous real-time kinematics (SRTK) and asynchronous real-time kinematics (ARTK).

The term $\delta \varphi^{i}$ in Equation (1) can be ignored here since it is relatively stable over a 15 min step-size [21] and $\delta \varphi_{B}$ between satellites can be eliminated via the single-difference method. ARTK methods are different from SRTK methods because the former must deal with several errors separately before forming DD observations, as shown in Figure 1.

This study details how, in contrast to the broadcast ephemeris used in the ARTK method [13], the satellite clock error $c\delta t^{ij}\left(T_{0},T_{1}\right)$ and the ephemeris error $E^{ij}\left(T_{0},T_{1}\right)$ can be eliminated in the new method by using the final precise ephemeris for post-processing or the ultra-rapid precise ephemeris for real-time processing. In addition, the tropospheric delay is corrected via the Saastamoinen model with standard atmosphere. To realize a high precision, such as centimeter-level positioning, using data from GNSS, it is essential that the ionospheric-induced error be well understood [22,23,24,25]. This is especially true for the asynchronous model in which it is difficult to eliminate this error. For this reason, the influence of ionospheric delay and the residual error in the asynchronous model are explored in detail.

The nonlinear Equations (1)–(3) were converted into linear equations using a Taylor expansion in preparation for least squares estimation. For example, the linear equation that corresponds to Equation (3) is as follows:

(4) $$\left[\begin{array}{c} v_{BA}^{ij}\left(t_{0},t_{1}\right)\\ \ldots \\ v_{BA}^{ik}\left(t_{0},t_{1}\right)\\ \ldots \\ v_{BA}^{il}\left(t_{0},t_{1}\right) \end{array}\right]=\left[\begin{array}{cccccc} (\frac{\mathrm{dx}}{\mathrm{ds}}\;\frac{\mathrm{dy}}{\mathrm{ds}}\;\frac{\mathrm{dz}}{\mathrm{ds}}{)}_{BA}^{ij} & \lambda & 0 & 0 & 0 & 0\\ \ldots & 0 & \ldots & 0 & 0 & 0\\ (\frac{\mathrm{dx}}{\mathrm{ds}}\;\frac{\mathrm{dy}}{\mathrm{ds}}\;\frac{\mathrm{dz}}{\mathrm{ds}}{)}_{BA}^{ik} & 0 & 0 & \lambda & 0 & 0\\ \ldots & 0 & 0 & 0 & \ldots & 0\\ (\frac{\mathrm{dx}}{\mathrm{ds}}\;\frac{\mathrm{dy}}{\mathrm{ds}}\;\frac{\mathrm{dz}}{\mathrm{ds}}{)}_{BA}^{il} & 0 & 0 & 0 & 0 & \lambda \end{array}\right]\left[\begin{array}{c} \Delta x\\ \Delta y\\ \Delta z\\ \Delta \nabla N_{B,A}^{i,j}\\ \ldots \\ \Delta \nabla N_{B,A}^{i,k}\\ \ldots \\ \Delta \nabla N_{B,A}^{i,l} \end{array}\right]-\left[\begin{array}{c} l_{BA}^{ij}\left(t_{0},t_{1}\right)\\ \ldots \\ l_{BA}^{ik}\left(t_{0},t_{1}\right)\\ \ldots \\ l_{BA}^{il}\left(t_{0},t_{1}\right) \end{array}\right].$$

Equation (4) can be abbreviated further as:

(5) $$V=A_{\mathrm{matrix}}X-L,$$

and

(6) $$\begin{array}{ll} l_{BA}^{ij}\left(t_{0},t_{1}\right)= & \varphi_{BA}^{ij}\left(t_{0},t_{1}\right)-R_{BA}^{ij}{\left(T_{0},T_{1}\right)}_{0}-\lambda N_{BA}^{ij}\left(t_{0},t_{1}\right)-c\delta t^{ij}\left(T_{0},T_{1}\right)-E^{ij}\left(T_{0},T_{1}\right)\\ & \;-T_{BA}^{ij}\left(t_{0},t_{1}\right)+I_{BA}^{ij}\left(t_{0},t_{1}\right)-\varepsilon_{BA}^{ij}\left(t_{0},t_{1}\right), \end{array}$$

where V represents the observed residual values, $A_{\mathrm{matrix}}$ is the coefficient matrix of the estimated parameter, X is the parameter to be estimated, L is a constant term vector, and $R_{BA}^{ij}{\left(T_{0},T_{1}\right)}_{0}$ is the notation of approximate distance. The coefficient of the ambiguity parameter is the wavelength of the observed value; the coefficients of the coordinate parameters are expressed as $\left\{\frac{\mathrm{dx}}{\mathrm{ds}},\frac{\mathrm{dy}}{\mathrm{ds}},\frac{\mathrm{dz}}{\mathrm{ds}}\right\}$, where dx, dy, and dz are the difference of coordination between the rover receiver and satellite in three directions; and ds is the geometric distance between the rover receiver and the satellite. In both the undifferenced (Equation (1)) and DD (Equations (2) or (3)) equations, $A_{\mathrm{matrix}}$ is only related to the rover receiver and satellites and is not affected when the base receiver is interrupted. In contrast, L is different for the synchronous and asynchronous models. The most influential terms, namely the asynchronous ionospheric delay and the residual error, are discussed in more detail in the following sections.

### 2.2. Asynchronous Ionospheric Delay

To aid the study of the effect of ionospheric delay on the ARTK method, Equation (3) can be simplified as follows:

(7) $$\varphi_{BA}^{ij}\left(t_{0},t_{1}\right)=P_{BA}^{ij}\left(T_{0},T_{1}\right)-I_{BA}^{ij}\left(t_{0},t_{1}\right)+\varepsilon_{BA}^{ij}\left(t_{0},t_{1}\right),$$

where $P_{BA}^{ij}\left(T_{0},T_{1}\right)$ includes all items, except ionospheric delay, that can be eliminated in synchronous RTK, as it is approximately equal between the rover and base receivers. This is particularly true of and widely used in traditional RTK when baselines are short, which is also the focus of this study [26]. The ionospheric delay is defined as follows:

(8) $$I_{B}^{ij}\left(t_{0}\right)=I_{A}^{ij}\left(t_{0}\right).$$

Based on this:

(9) $$I_{BA}^{ij}\left(t_{0},t_{1}\right)=I_{A}^{ij}\left(t_{1}\right)-I_{B}^{ij}\left(t_{0}\right)=I_{A}^{ij}\left(t_{1}\right)-I_{A}^{ij}\left(t_{0}\right)=I_{A}^{ij}\left(t_{0},t_{1}\right).$$

Equation (9) shows that as long as the ionospheric delay variation on the rover receiver is known, the residual ionospheric delay in the asynchronous RTK can be computed. Fortunately, the observations of the rover receiver are available and the ionospheric delay of the single-difference observation L1 frequency of the rover receiver can be derived from the original dual-frequency observations value [22]:

(10) $$I_{A_{L1}}^{ij}\left(t_{1},t_{0}\right)=\frac{f_{2}^{2}}{f_{2}^{2}-f_{1}^{2}}\left(\lambda_{1}\varphi_{A_{L1}}^{ij}\left(t_{0},t_{1}\right)-\lambda_{2}\varphi_{A_{L2}}^{ij}\left(t_{0},t_{1}\right)\right).$$

**Figure 2:**
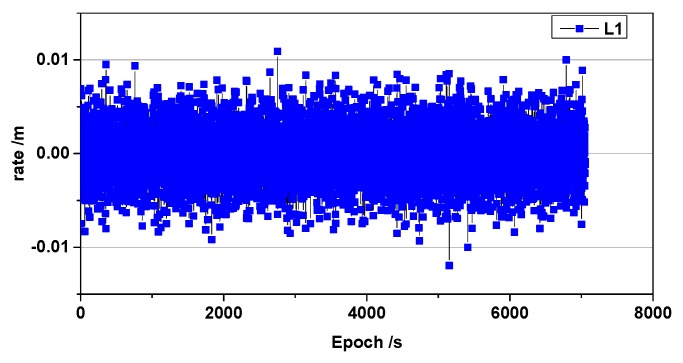
Changes in the ionospheric delay rate of the BeiDou C10 satellite.

In the following, $I_{A_{L1}}^{ij}\left(t_{1},t_{0}\right)$ can be denoted simply as ΔI. We analyzed the ionospheric delay rate using data published by Liu (2009), which described the ionospheric total electron content (TEC) rate. The results of the analysis are shown in Figure 2. It is shown that the test environment is ionospheric quiet.

**Figure 3:**
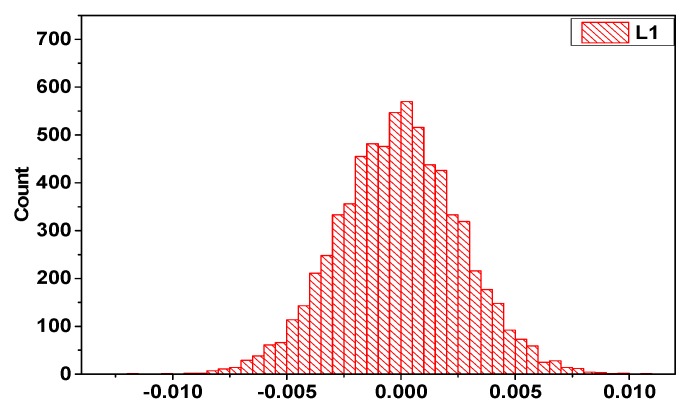
Histogram of BeiDou C10 satellite ionospheric delay rate changes.

Figure 3 shows that the ionospheric delay rate resembles the standard normal distribution near zero, which is consistent with the results of previous studies. This characteristic can be used for cycle slip detection [22,27].

**Figure 4:**
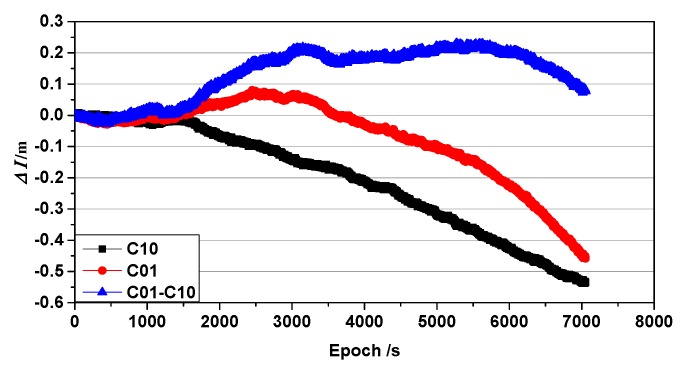
Accumulated ionospheric delay of C01 and C10 L1 satellite frequencies.

In Figure 3, the curve is shifted slightly to the left of zero, which introduces a bias into the accumulated ionospheric delay. This bias is shown in Figure 4. As the accumulated ionospheric delay variations of each satellite are similar, only C01 and the reference satellite C10 were used for this explanation.

The cumulative ionospheric delay variations of BeiDou satellites C01 and C10 are shown in Figure 4, where the blue line represents the single-difference between the C01 and C10 satellites with satellite C10 as the reference. As shown, the ionospheric delay accumulation does not change linearly and gradually deviates away from zero. A similar deviation arises when the base receiver is interrupted; if this deviation is not corrected, it will cause the positioning results to deviate from the true value. Note that the cycle slip in this study was corrected using other algorithms [28].

### 2.3. Asynchronous Residual Error

Based on the ionospheric delay variations detailed in the previous section, Figure 4 shows a comparison of the constant terms L for the C01 satellite for the SRTK and ARTK methods before and after ionospheric delay correction, with the C10 satellite as the reference satellite. The blue line is the result of SRTK, the values from which serve as the reference. The black line represents the traditional ARTK result, while the red line represents the ARTK result after corrections for ionospheric delay.

**Figure 5:**
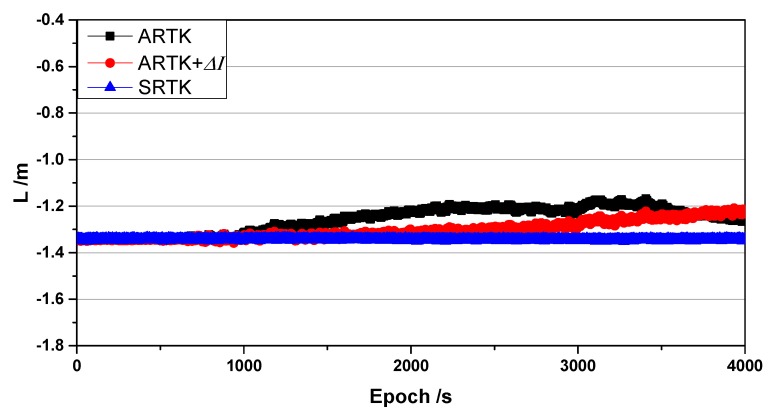
Asynchronous constant term before and after ionospheric delay compensation.

The residual error in the constant term L between the SRTK and ARTK algorithms after compensation for the ionospheric delay displays a distinctly gradual linear change over a short period of time, as shown in Figure 5. Without compensation for the ionospheric delay it is difficult to predict. It is denoted ΔL=L(ARTK)−L(SRTK) (the black line) and ΔL=L(ARTK+ΔI)−L(SRTK) (the red line), that is, the difference between the proposed algorithm ARTK and SRTK. This difference can be modeled in the time domain. A first-degree polynomial function is a straightforward option for predicting the difference and can be represented as:

(11) $$\Delta L_{i}=a_{0}+a_{1}t_{i}+\varepsilon_{i}\;\;\;\left(0\leq i\leq n\right),$$

where $t_{i}$ is the epoch time, $a_{0}\;\mathrm{and}\;a_{1}$ are the parameters of the model, $\varepsilon_{i}$ is the model error, and n is the window size. Considering the real-time performance and stability required by the calculations and parameter estimation, the size of n in this study was set to 10 min for 1 Hz data and 15 min for 1/30 Hz data, respectively, based on the results of the experiments.

In this study, the above derivation was employed to correct the asynchronous errors generated in the asynchronous model when the base receiver was interrupted.

### 2.4. New ARTK Strategy

**Figure 6:**
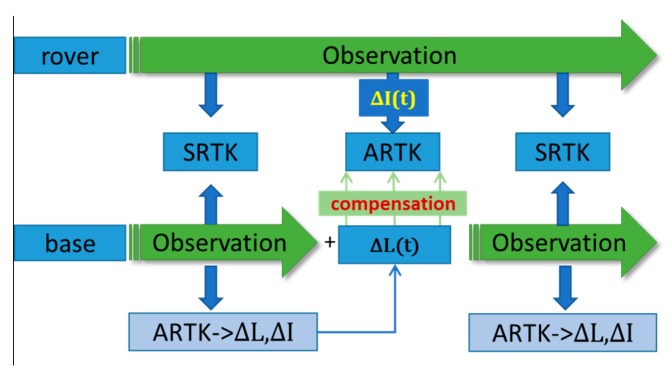
Modified asynchronous real-time kinematics strategy that considers ionospheric delay.

As the traditional SRTK method is relatively mature and well understood, the purpose of this section is to describe the ARTK method with asynchronous residual error compensation. Figure 6 shows that when the base receiver is available, both the SRTK and ARTK methods are computed in parallel. The SRTK method is used to output the solution results, whereas the ARTK method is used to generate asynchronous residual errors ΔL and ΔI. When the base receiver is unavailable, ΔL and ΔI can be generated using the pre-sequence epoch value to generate the term ΔL(t) and ΔI(t) using the observations of the rover receiver. These terms can be used to compensate for the asynchronous bias in the asynchronous model. The normal solution can then be calculated as per the traditional SRTK method. When the base station is back online, the parallel computation between the SRTK method and ARTK method resumes.

## 3. Experimental Validations

**Figure 7:**
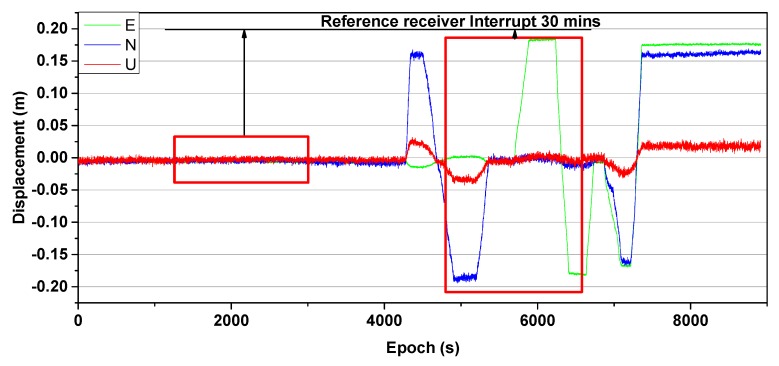
Three directions, E, N, and U, of the experimental process sequence diagram and the displacement along these directions across the epochs.

To demonstrate the validity of the proposed method for evaluating asynchronous errors in the asynchronous model during the period that a base station is interrupted, a dataset was obtained via simulation. A base station and rover receivers (UNICORECOMM-UR380, UNICORECOMM, China) were used to collect dual-frequency BeiDou data over a period of 2.5 h with a sampling interval of 1 s and a short baseline of approximately 30 m. One receiver was placed on a static platform to act as the base station receiver, while the other was mounted on an experimental mobile platform used to simulate deformation. Once the static data were collected (around the 4000th epoch), a dynamic simulation was performed using the experimental platform (about after the 4000th epoch). Eight BeiDou satellites were available during the period in which the data were collected. An SRTK solution sequence in the East, North and Up (E, N, and U) directions that had no missing data was considered reliable even though the actual data values were not obtained. A representative data sequence is shown in Figure 7.

The red boxes in the Figure 7 present an interruption of 30 min at the base receiver. The SRTK method can be considered reliable as the assumed interruption is similar to an actual data interruption. The proposed method was validated in two stages, static and dynamic, using the following three schemes.

**Scheme 1:** The SRTK method is used and provides reliable values.

**Scheme 2:** The new ARTK method that employs the final satellite orbits and clocks provided by the GeoForschungsZentrum (GFZ) Centre for Geosciences for post-processing is used.

**Scheme 3:** The new ARTK method that employs the ultra-rapid satellite orbits and clocks provided by the analysis center at Wuhan University for real-time processing is used.

### 3.1. Static Positioning Experiment

**Figure 8:**
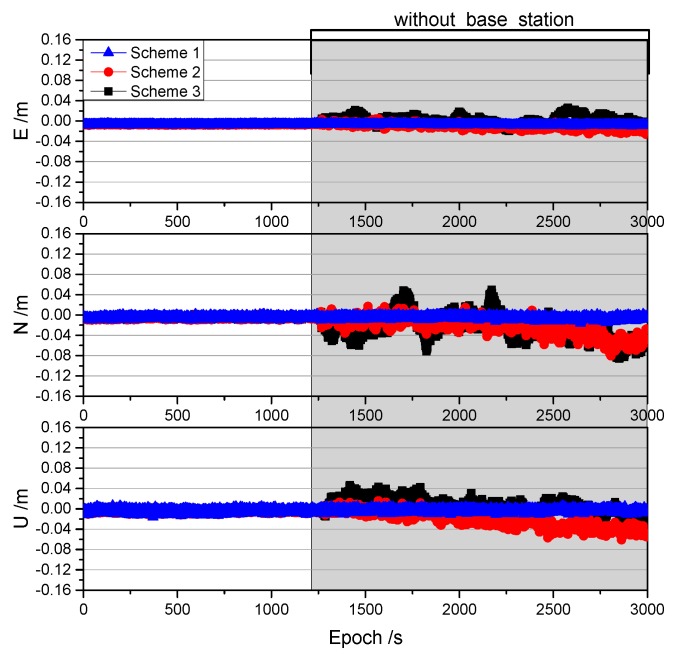
Comparison of the results obtained from three different schemes in the static experiment.

To evaluate the performance of the proposed method, we simulated a 30 min interruption at the base receiver when the rover receiver was static. A comparison of the results obtained for the three schemes over the 30 min period is shown in Figure 8.

As shown in Figure 8, Schemes 2 and 3 achieved centimeter-level positioning accuracy in the directions of E, N, and U using the correction provided by the proposed method within 30 min of being interrupted. The positioning accuracy of Schemes 2 and 3 is shown in Table 1.

The positioning accuracy of Scheme 2 was found to be better than that of Scheme 3, and millimeter-level accuracy was achieved within 10 min of the interruption. The standard deviation of the Scheme 3 is only smaller than that of the Scheme 2 (only 1 mm) in the U direction after the interruption of 30 min. Despite this, it is also clear that Scheme 2 is better in the total error of the three directions of the ENU. The positioning accuracy in the E direction was the smallest as the configuration of the geosynchronous equatorial orbit satellites was such that they were distributed in the east-west direction in the BeiDou navigation satellite system [13]. The accuracy in the U direction was the first to be affected by systematic errors because the vertical dilution of precision (VDOP) was larger than the horizontal dilution of precision, and the error was projected mostly in the height component [13]. It can also be seen from Figure 8 that the noise in the N direction was the largest, especially for Scheme 3. Moreover, there appeared to be a periodic duration at 5 min intervals in Scheme 3, which is consistent with the sampling rate represented by the clock file used in this study. To address this, we down-sampled the 30 s clock file of Scheme 2 to 5 min (that only use the epoch of clock data that can be divisible by 5 min, such as 0, 5, 10, 15 min, etc.) and obtained the same results as in Scheme 3. This suggests that if a clock file with a higher sampling rate is available, then the real-time accuracy will be improved [29,30].

### 3.2. Dynamic Positioning Experiment

**Figure 9:**
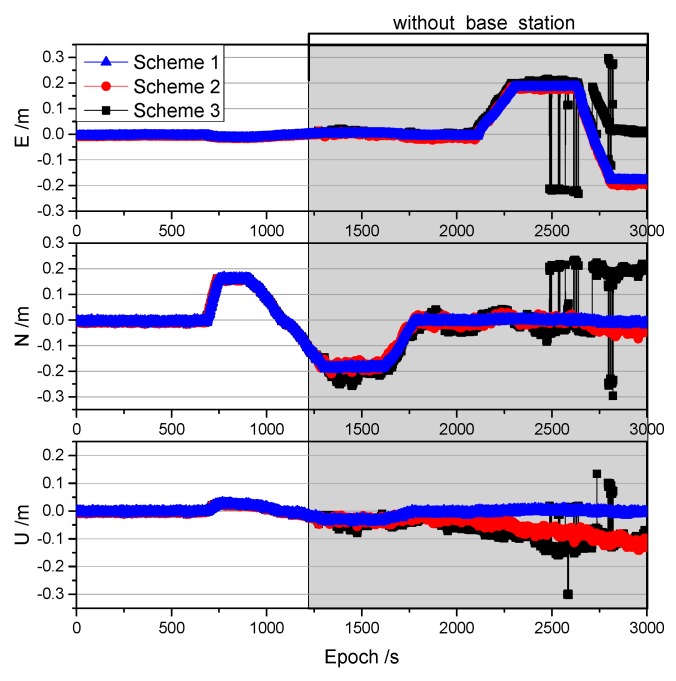
Comparison of the results obtained from the three schemes used in the dynamic experiment.

In the dynamic phase (although the speed of the antenna is approximately constant during the motion, it is still dynamic since its state has changed), the proposed method was validated using Schemes 2 and 3 for post-processing and real-time applications, respectively. In this phase, a base receiver with a 30 min interruption was simulated. The results of this test are shown in Figure 9 and Table 2.

Figure 9 shows that the results of the dynamic positioning experiment were worse than those of the static experiment, which is consistent with the common understanding that the quality of dynamic observations is significantly worse than those obtained via static observations [31]. As in the static tests, the U direction was more susceptible to systematic errors due to the large VDOP value [13]. In addition, the results obtained from Scheme 3 were not as good as those of Scheme 2, as the sampling rate in the clock file was 5 min. A number of significant errors were noted in the results of Scheme 3 even when the interruption time approached 20 min, and its deviation increased significantly after 20 min of interruption, as shown in Table 2. It was evident that the U directions of Schemes 2 and 3 were more affected by the systematic error than they were in the static experiment. Fortunately, the impact of the systematic error was relatively insignificant in the first 10 min after the interruption and little impact was observed on the horizontal direction throughout the 30 min interruption time span. 

**Figure 10:**
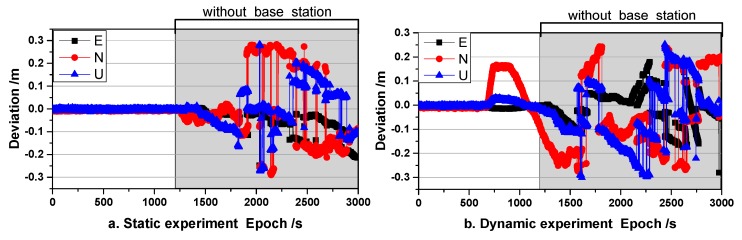
Traditional ARTK result ((**a**) is a static experiment and (**b**) is a dynamic experiment).

The proposed method was shown to maintain the positioning service at centimeter-level accuracy for a time span of 15 min when the base receiver was unavailable, which is an improvement over previous studies that demonstrated only a maximum 3 min of availability [11,12,13,14,15,16]. Especially compared to traditional ARTK (traditional ARTK result is shown in Figure 10), the proposed method takes these asynchronous errors into account.

In general, in the static experiments, both real-time and post-processing scenarios were able to achieve centimeter-level accuracy within a 30 min interruption, as shown in Table 3. In contrast, for the dynamic experiment, centimeter-level accuracy was achieved horizontally within 30 min and vertically within 18 min in the post-processing scenario. Even in the U direction with a large projected system error, the centimeter-level accuracy could be maintained for approximately 15 min in the real-time processing scenario.

### 3.3. Results for Different Short Baseline Lengths

**Figure 11:**
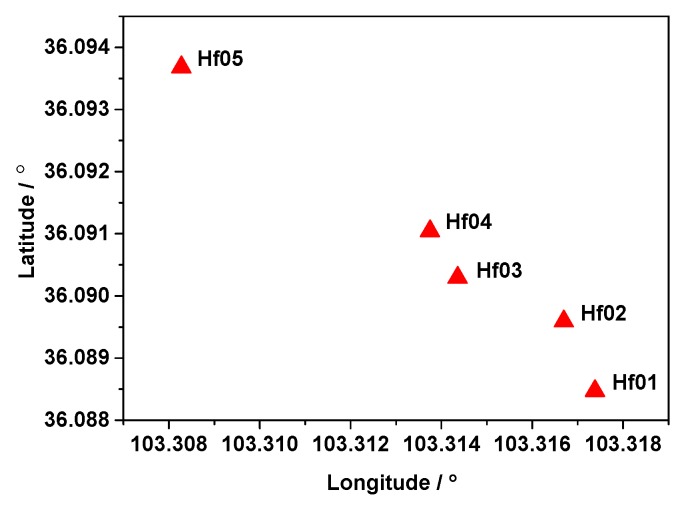
Layout of the stations used in the experiment.

**Figure 12:**
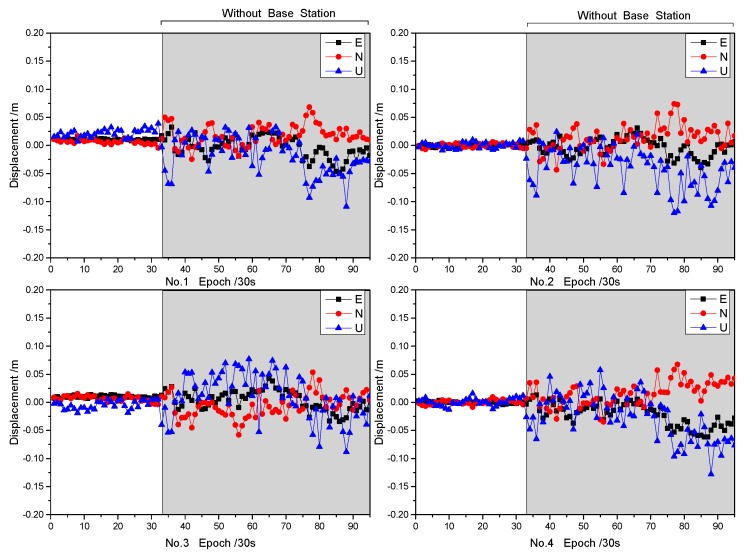
Displacement of the proposed ARTK method on baselines No. 1 to 4.

To further demonstrate the validity of the proposed procedure for significance testing of asynchronous errors, four datasets (shown in Table 4) were analyzed. Dual-frequency BeiDou data with a sampling interval of 30 s were collected at the same time from a landslide monitoring station in Gansu Province, China. As mentioned in the static experiment, if the clock file sampling rate is consistent, the results of Scheme 2 and Scheme 3 are consistent. Therefore, in this section, we use only ultra-rapid products to verify the proposed method. The Station Hf01 is located in the stable region as the base station (Figure 11). The deviations of the proposed ARTK method on baselines No. 1 to 4 are shown in Figure 12 and Table 5.

Figure 12 and Table 5 show that the proposed method can maintain the positioning accuracy of centimeters or centimeter-level for a short period after interruption. Within a short period after interruption, the reason for the large error is mainly due to the increase of the noise of the observations, as shown in Equations (4)–(6). Of course, with the passage of time, systematic deviations such as No. 4 are inevitable. Therefore, the deviation that occurs after 15 min cannot be distinguished from the actual displacement. In practical applications, the results are considered reliable within 15 min after the interruption.

## 4. Discussion and Conclusions

The present study proposed a new ARTK positioning method intended to provide continuous rover positioning even when the base station is interrupted and leads to gaps in the obtained data. This is accomplished via an optimal method of handling asynchronous errors, including errors related to the satellites and the atmosphere. The asynchronous ionospheric delay was corrected by carrier phase measurements of the rover receiver and the asynchronous residual error was predicted via a first-degree polynomial function. The results of the experiments (collected for 2 h of data) confirmed that the proposed method could achieve centimeter-level accuracy when the time span of the interruption lasted up to 15 min, compared with previous studies (no more than 3 min). The accuracy in the vertical direction decreased faster as the interruption time increased, but was still at the centimeter-level for interruptions up to 15 min. This method can be used to mitigate problems with base receiver interruption by correcting asynchronous errors and is expected to be sufficiently reliable to expand the continuity and availability of RTK technology used to monitor deformations. However, this method does not eliminate asynchronous errors completely; the remaining errors can cause deviations in the positioning results and these might gradually increase in magnitude with the passage of time. Future research will be aimed at improving the positioning accuracy of the proposed method by an in-depth study of the impact of asynchronous errors to overcome these deviations.

## Figures and Tables

**Table 1:** Standard deviation of the results obtained from the static experiment.

Time Span of the Interruption (min)	Standard Deviation (m) of Scheme 2			Standard Deviation (m) of Scheme 3		
	E	N	U	E	N	U
**10**	0.003	0.009	0.007	0.005	0.024	0.011
**20**	0.004	0.011	0.011	0.007	0.023	0.013
**30**	0.005	0.017	0.016	0.008	0.025	0.015

**Table 2:** Standard deviation of the results obtained from the dynamic experiment.

Time Span of the Interruption (min)	Standard Deviation (m) of Scheme 2			Standard Deviation (m) of Scheme 3		
	E	N	U	E	N	U
**10**	0.005	0.011	0.010	0.006	0.019	0.013
**20**	0.005	0.011	0.025	0.008	0.023	0.034
**30**	0.008	0.017	0.041	0.095	0.095	0.049

**Table 3:** Summary of the time span over which centimeter-level accuracy (standard deviation <3 cm) was maintained in this experiment (unit: min).

	Post-Processing			Real-Time Processing		
	E	N	U	E	N	U
**Static**	30	30	30	30	30	30
**Dynamic**	30	30	18	20	20	15

**Table 4:** Data description of the four datasets.

Dataset	Stations	Receiver Type	Antenna Type	Length
No. 1	Hf01–Hf02	UR380	HG-GOYH7151	139 m
No. 2	Hf01–Hf03	UR380	HG-GOYH7151	225 m
No. 3	Hf01–Hf04	UR380	HG-GOYH7151	311 m
No. 4	Hf01–Hf05	UR380	HG-GOYH7151	884 m

**Table 5:** Standard deviation of the results obtained from baselines No. 1 to 4. The interruptions lasted 15 and 30 min (unit: m).

		15 min			30 min	
	E	N	U	E	N	U
**No. 1**	0.014	0.019	0.029	0.019	0.019	0.035
**No. 2**	0.012	0.019	0.028	0.012	0.023	0.037
**No. 3**	0.012	0.019	0.035	0.018	0.021	0.041
**No. 4**	0.013	0.018	0.029	0.021	0.022	0.041
